# Linking Sustainable Smart Food Packaging to Healthy Eating Behaviors: A TPB–Perceived Value Framework with IPMA Analysis

**DOI:** 10.3390/foods15091496

**Published:** 2026-04-25

**Authors:** Juncheng Mu, Linglin Zhou, Chun Yang

**Affiliations:** 1 School of Fine Arts, Nanjing Normal University, Nanjing 210023, China; 211601009@njnu.edu.cn; 2 School of Design, Anhui Polytechnic University, Wuhu 241000, China; zll@ahpu.edu.cn; 3 School of Design, Jiangnan University, Wuxi 214122, China

**Keywords:** smart food packaging, perceived value theory, theory of planned behavior, purchase intention, healthy eating behavior

## Abstract

Driven by the iteration of digital technologies and the upgrading of residents’ health consumption demands, smart food packaging has developed rapidly and is widely applied across various food categories. However, issues such as consumer cognitive biases and insufficient acceptance hinder its market penetration. This paper constructs a chained mediation model based on the Theory of Planned Behavior (TPB) and Perceived Value Theory, employing PLS-SEM and IPMA methods to validate multiple research hypotheses. It innovatively integrates multiple theories to establish an interdisciplinary research framework, overcoming the limitations of single theories. The analysis, combined with IPMA, clarifies the priority of each variable, addressing existing research gaps. The results indicate that the four perceptual factors of smart food packaging significantly and positively influence the three core constructs of TPB, with experiential factors exerting the strongest drive on individual needs. The TPB constructs significantly and positively affect perceived value, perceived trust, and self-efficacy, with the drive of individual needs being most prominent. Perceived trust has the strongest influence on healthy eating behavior. IPMA analysis reveals that perceived value (PV) is a key area for improvement, while individual needs (IN) and self-efficacy (SEHB) are key areas of strength. This study elucidates the internal mechanisms through which smart food packaging influences consumers’ healthy eating behaviors, providing theoretical and practical support for enterprises to optimize design and guide healthy consumption.

## 1. Introduction

Driven by the dual forces of digital technology iteration and the upgrading of residents’ health consumption demands, smart food packaging, as a core direction for the transformation and upgrading of the traditional packaging industry, is breaking through the functional limitations of traditional packaging and becoming an important bridge connecting food enterprises, products, and consumers [1]. It integrates modern information technologies such as sensors, radio-frequency identification (RFID), and digital labels with packaging materials and structural design, possessing characteristics of information interaction, status monitoring, and functional response [2,3,4,5]. Its core value lies in transcending the physical protection and basic information display functions of traditional packaging to achieve diversified services such as visualized traceability throughout the food lifecycle, real-time quality monitoring, and personalized health guidance [6]. While existing research has confirmed the significant roles of smart food packaging in safeguarding food safety and enhancing consumer experience [7,8,9], it has predominantly concentrated on technological development and functional realization. There remains a scarcity of systematic investigation into the underlying mechanisms by which intelligent packaging influences consumers’ psychological cognitions and behaviors, particularly lacking analyses from a multi-theoretical integration perspective [10]. Currently, the smart food packaging industry is experiencing rapid growth, yet market penetration is hindered by prominent issues such as insufficient consumer awareness and weak purchase intentions [1,11]. This significant challenge renders existing findings inadequate for effectively guiding enterprises in optimizing design and addressing market pain points. Therefore, a thorough exploration of the impact mechanisms of its perceived characteristics on consumer behavior holds substantial theoretical and practical significance.

To this end, this study integrates the Theory of Planned Behavior (TPB) [12] and Perceived Value Theory [13] to construct a theoretical framework for the influence of perceived intelligent food packaging on consumers’ purchase intentions and healthy eating behaviors. Within TPB, consumer behavioral intention is jointly determined by attitude, subjective norm, and perceived behavioral control [14]; in Perceived Value Theory, consumers’ value judgments of intelligent packaging serve as the core link connecting their perceptions to behavioral intentions [15,16]. This study conceptualizes the core constructs of TPB as internal psychological factors and the perceived characteristics of intelligent food packaging as external stimuli, investigating the indirect and direct effects of external stimuli on consumer behavior through internal psychological factors. The objective is to elucidate the mechanism by which intelligent food packaging affects consumers’ purchase intentions and healthy eating behaviors, thereby constructing a complete logical chain of “external stimuli—internal cognition—value judgment—behavioral intention” and providing interdisciplinary theoretical support for this field.

The core innovation of this research lies in addressing the existing research gaps: traditional studies either independently examine the functions of intelligent packaging from a technical perspective, neglecting consumers’ psychological cognitive patterns [17,18], or analyze consumer acceptance intentions based on a single theory [19], lacking multi-dimensional interactive analysis of technological perception, value judgment, and psychological motivation. Through multi-theoretical integration, this study links the internal psychological variables of TPB with the external variables of intelligent packaging perception, filling the void in single-theory explanations of the “technological stimulus—consumer decision-making” conversion mechanism. It clarifies the pathways through which different dimensions of intelligent packaging indirectly influence consumer behavior by affecting the core constructs of TPB, thereby constructing a model of multi-variable synergistic influence. Concurrently, it offers practical guidance for optimizing intelligent packaging design and promotion strategies, enhancing consumer acceptance, and fostering healthy eating behaviors. The structure of this paper is as follows: Introduction, Relevant Theoretical Foundations, Research Methodology Design, Empirical Analysis, Research Discussion, and Conclusion and Limitations.

## 2. Literature Review

### 2.1. Theory of Planned Behavior

The Theory of Planned Behavior (TPB) is a central theoretical framework in behavioral science for predicting and explaining individual behavior. Developed by Ajzen as an extension of the Theory of Rational Behavior (TRA), its core logic posits that individual behavioral decisions are directly driven by behavioral intention, which in turn is jointly determined by three key constructs: attitude, subjective norm, and perceived behavioral control [12]. The core constructs are clearly defined with distinct boundaries: Attitude refers to an individual’s positive or negative evaluation of a specific behavior, stemming from beliefs about its outcomes and value judgments. Subjective norm reflects an individual’s perception of the expectations and approval from significant others or social groups regarding the performance of that behavior. Perceived behavioral control, conversely, indicates an individual’s subjective judgment of their ability, resources, and the extent of control required to perform the behavior [12].

The TPB theory, owing to its explanatory power and generalizability, is widely applied in various research domains, including consumer behavior and health management. In consumer behavior research, this theory is frequently employed to analyze the acceptance intention and purchase decision mechanisms for novel food products and smart devices [20]. In the realm of health behaviors, it serves as a classic model for explaining individuals’ choices in healthy lifestyles, such as diet and exercise [21,22]. The adaptability of TPB is particularly prominent for food smart packaging, an emerging technological product: consumers’ attitudes toward smart packaging directly reflect their subjective evaluation of its functional value; the consumption atmosphere and peer recommendations in the social environment correspond to the role of subjective norms; and consumers’ control over operating smart packaging and accessing information constitutes the core content of perceived behavioral control [14]. Existing research has integrated external factors such as the functional characteristics of packaging and social influences with TPB constructs, confirming that functions of smart packaging, like freshness monitoring and traceability, can enhance consumer attitudes and subsequently influence purchase intentions [14,15,23]. Furthermore, advocacy for healthy and environmentally friendly consumption within the social environment can positively drive consumers’ acceptance intention of smart packaging through subjective norms [24]. Concurrently, perceived behavioral control, acting as a bridge between technological characteristics and consumer behavior, directly determines whether consumers can successfully use smart packaging and translate it into actual behavior [25].

Within the traditional Theory of Planned Behavior, perceived behavioral control emphasizes external resources and self-efficacy judgments, offering relatively limited explanation for intrinsic motivational aspects [12]. In scenarios involving healthy consumption and the application of smart technologies, individual needs, as sources of intrinsic motivation, often exert a more critical and direct influence on behavioral intentions [26]. Individual needs not only encompass an intrinsic desire for the value of a behavior but also, to a certain extent, integrate and transcend the controllability judgments of traditional perceived behavioral control, achieving a theoretical upgrade from “feasibility judgment” to “motivational drive.” Therefore, this study introduces individual needs into the TPB model to more comprehensively elucidate the psychological mechanisms and behavioral regularities of consumers in the context of smart packaging usage.

### 2.2. Factors of Smart Food Packaging

As a digitally upgraded form of traditional packaging, the core value of smart food packaging transcends the basic functions of physical protection and preservation inherent in traditional packaging. Instead, it pivots towards diverse services encompassing information interaction, intelligent monitoring, health guidance, and environmental sustainability [6]. Integrating existing research with the theoretical framework of this study, the perceptual characteristics of smart food packaging can be systematically deconstructed into four core dimensions: informational factors [27,28], environmental factors [29,30], functional factors [28], and experiential factors [28,29]. Each dimension operates independently while collectively forming an external stimulus system that influences consumer psychological cognition and behavioral decisions [28].

Information factors focus on the content, delivery efficiency, and transparency of packaging information [2,3,4,5], encompassing the entire food lifecycle. Leveraging digital technologies addresses the pain points of traditional labeling [27,28], directly influencing consumer trust in food safety and health perceptions. These factors serve as critical stimuli impacting attitudes and subjective norms [31,32]. Environmental factors align with the “dual carbon” strategy and sustainable consumption trends [29,30]. The core lies in the environmental friendliness and sustainability of packaging materials [33]. Consumer perceptions include material recognition and corporate philosophy alignment, resonating with the green orientation in healthy consumption [34,35], and influencing subjective norms and social value judgments. Functional factors are what distinguish smart packaging from traditional packaging [28], encompassing two main types: status monitoring and active release [7,8,9,36,37]. These directly reflect actual utility value and are the core support for consumers forming positive attitudes and enhancing perceived value [14]. Experience factors emphasize consumer interaction and feelings [28,29], including operational convenience and interface friendliness [38]. These directly correspond to the core tenets of perceived behavioral control within the TPB, influencing consumers’ sense of control and psychological acceptance [14,34].

### 2.3. Perceived Value Theory

Perceived value theory is a core theory in consumer behavior that explains the “external stimuli → internal cognition → behavioral transformation” process. Pioneered by Zeithaml, its central tenet is the consumer’s comprehensive evaluation and trade-off between perceived gains and perceived losses during product acquisition and use [13]. This involves a holistic assessment of perceived benefits (e.g., functional, emotional, health-related gains) [39,40] versus perceived costs (e.g., monetary, temporal, psychological risks) [41]. The underlying logic is that external stimuli (such as product characteristics and technological functions) influence consumers’ value perceptions, altering their overall product evaluation and subsequently driving purchase intentions and actual behavior [42,43]. Unlike the TPB’s focus on the “attitude, norm, control” logic of behavioral decision-making, perceived value theory centers on consumers’ subjective trade-offs and value judgments, providing the foundational logic for explaining why consumers accept a particular product or develop behavioral intentions [13]. This complementarity with the TPB’s emphasis on behavioral decision logic makes it a critical supporting framework for explaining consumer acceptance of smart packaging.

In the context of food smart packaging research, perceived value plays a crucial mediating role in the relationship between smart packaging and consumer behavior. For instance, features like freshness monitoring and traceability in smart packaging can significantly enhance consumers’ perceived functional value. The use of environmentally friendly materials can bolster their perceived social and emotional value. Furthermore, a convenient interactive experience can reduce perceived losses, ultimately elevating overall perceived value [27]. Concurrently, perceived value directly links the external perceptual characteristics of smart packaging to consumer purchase intentions and healthy eating behaviors, serving as the pivotal nexus connecting TPB’s psychological constructs with behavioral outcomes [44,45,46]. Therefore, perceived value theory addresses the explanatory gap in value trade-offs within the TPB and forms a key component of the theoretical integration in this study.

### 2.4. Theoretical Logic

The theoretical framework constructed in this study centers on the interplay between the four perceptual elements of smart food packaging and the three core constructs of the TPB, transmitting through perceived value, perceived trust, and self-efficacy for healthy behavior to behavioral outcomes. These four elements do not operate independently but rather interact through multiple cross-pathways influencing the three core TPB constructs, thereby forming a comprehensive driving matrix.

**Figure 1:**
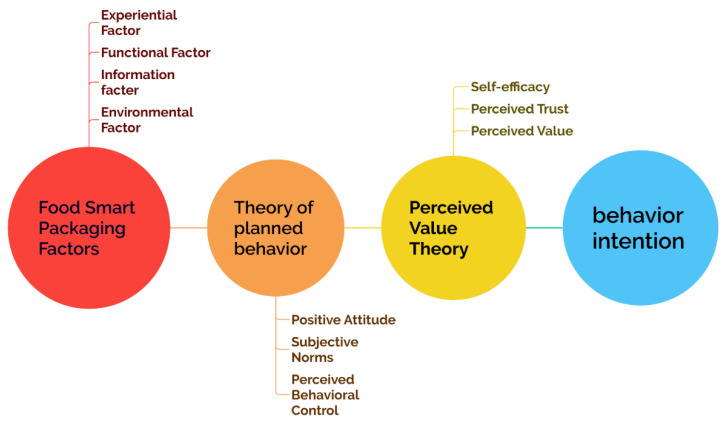
Theoretical logic diagram.

As illustrated in Figure 1, after the four perceptual elements activate the three core TPB constructs, consumers’ psychological cognitions transform into perceived value, perceived trust, and self-efficacy for healthy behavior. These three act as mediating variables to drive the formation of purchase intention, subsequently influencing healthy eating behavior, thus completing a closed-loop cycle of “external stimulus—psychological cognition—value judgment—actual behavior.” This integrated logic transcends the linear association model of “single element, single construct” found in traditional research, revealing the deep mechanism by which the four elements holistically empower consumer psychology. This not only strengthens the explanatory power of TPB in emerging technology consumption scenarios but also provides a systematic theoretical basis for intervening in consumer psychology and behavior through optimized smart packaging design, thereby fully supporting the core hypotheses and model architecture of this study.

## 3. Research Hypotheses and Model

### 3.1. Research Hypotheses

#### 3.1.1. Influence of Perceived Smart Packaging Factors on Core Constructs of TPB

Information factors represent the core perceptual dimension of smart food packaging, utilizing digital labels, traceability technology, and other means to achieve transparent communication of food throughout its lifecycle, encompassing traceability information, nutritional content, freshness feedback, and more [2,3,4,5]. Within the framework of the Theory of Planned Behavior (TPB), information factors comprehensively influence consumers’ attitudes, perceptions of the social environment, and individual needs by mitigating information asymmetry and enhancing cognitive certainty: information transparency can strengthen consumers’ recognition of smart packaging’s food safety assurance, fostering positive attitudes; social-level information sharing and consensus building will reinforce a receptive atmosphere in the social environment towards smart packaging; and the experience of fulfilling personal information acquisition needs will enhance consumers’ perceived ability to use smart packaging.

Environmental factors embody the sustainable attributes of smart packaging, centering on bio-based degradable materials and low-carbon production processes, aligning with the “dual carbon” strategy and green consumption concepts [29,30]. From a TPB perspective, environmental factors influence consumers’ psychological cognitions through value identification and social demonstration effects: the application of environmentally friendly materials can strengthen consumers’ social value identification with smart packaging, forming positive attitudes; societal advocacy for green consumption and the demonstration by others will enhance positive normative perceptions of the social environment; and the experience of satisfying personal environmental demands will strengthen consumers’ sense of behavioral control in choosing smart packaging.

Functional factors are the core characteristics distinguishing smart packaging from traditional packaging, encompassing practical functions such as freshness monitoring, active preservation, and traceability inquiries, directly ensuring food safety and consumer convenience [28]. According to TPB theory, functional factors act upon consumers’ psychological constructs through perceived utility and capability empowerment: reliable functional performance can enhance consumers’ utility evaluations of smart packaging, forming positive attitudes; the safety assurance provided by functions will create a social-level reputation effect, reinforcing positive social norms; and the fulfillment of personal safety and health needs by functions will enhance consumers’ perceived behavioral control in using smart packaging.

Experiential factors focus on consumers’ interactive experiences with smart packaging, including operational convenience, interaction friendliness, and usage comfort, directly determining the usage threshold and experience satisfaction [28,29]. Within the TPB framework, experiential factors influence consumers’ psychological cognitions through emotional interaction and capability empowerment: a fluid interactive experience can elicit pleasure, strengthening positive attitudes toward smart packaging; a good usage experience will lead to word-of-mouth dissemination, enhancing the receptive atmosphere in the social environment; and convenient operation will lower the barrier to entry, strengthening consumers’ confidence in their ability to use smart packaging. Based on the aforementioned discussions, this paper proposes the following hypotheses:

**H1a.**  *Information factors of smart packaging have a positive influence on consumers’ attitudes toward smart packaging.*

**H1b.**  *Information factors of smart packaging have a positive influence on the social environment.*

**H1c.**  *Information factors of smart packaging have a positive influence on consumers’ individual needs regarding smart packaging.*

**H2a.**  *Environmental factors of smart packaging have a positive influence on consumers’ attitudes toward smart packaging.*

**H2b.**  *Environmental factors of smart packaging have a positive influence on the social environment.*

**H2c.**  *Environmental factors of smart packaging have a positive influence on consumers’ individual needs regarding smart packaging.*

**H3a.**  *Functional factors of smart packaging have a positive influence on consumers’ attitudes toward smart packaging.*

**H3b.**  *Functional factors of smart packaging have a positive influence on the social environment.*

**H3c.**  *Functional factors of smart packaging positively influence consumers’ individual needs for smart packaging.*

**H4a.**  *Experiential factors of smart packaging positively influence consumers’ attitudes towards smart packaging.*

**H4b.**  *Experiential factors of smart packaging positively influence the social environment.*

**H4c.**  *Experiential factors of smart packaging positively influence consumers’ individual needs for smart packaging.*

#### 3.1.2. Influence of TPB Core Constructs on Perceived Value Dimensions

Attitude represents a consumer’s overall positive or negative evaluation of smart packaging, stemming from their perceptions of its functional and experiential value [47]. According to perceived value theory, a positive attitude enhances consumers’ perceived benefits of smart packaging, thereby forming higher perceived value [48]. Concurrently, a positive attitude strengthens the perceived reliability of packaging information and functions, enhancing perceived trust [28]. Furthermore, an attitude that recognizes the value of smart packaging reinforces consumers’ confidence in achieving their healthy eating goals, thus improving health behavior self-efficacy [49]. The social environment reflects the expectations and acceptance of smart packaging by consumers’ perceived social groups [27]. From a perceived value perspective, a positive social environment strengthens social value recognition of smart packaging, increasing perceived value [50]. Social-level trust consensus enhances consumers’ perception of smart packaging reliability, thereby increasing perceived trust. Moreover, societal advocacy for healthy consumption reinforces consumers’ willingness to practice healthy eating through smart packaging, improving health behavior self-efficacy [51]. Consumers’ individual needs for smart packaging (perceived behavioral control) reflect their judgment of their own ability to use smart packaging and satisfy safety, health, and convenience needs [27]. According to perceived value theory, strong individual needs increase consumers’ perceived benefits of smart packaging, leading to higher perceived value. Satisfaction of needs enhances trust in smart packaging functions, thereby increasing perceived trust [1]. Confidence in one’s own control abilities further strengthens the sense of efficacy in achieving healthy eating goals through smart packaging.

**H5a.**  *Consumers’ attitudes towards smart packaging positively influence perceived value.*

**H5b.**  *Consumers’ attitudes towards smart packaging positively influence perceived trust.*

**H5c.**  *Consumers’ attitudes towards smart packaging positively influence health behavior self-efficacy.*

**H6a.**  *The social environment positively influences perceived value.*

**H6b.**  *The social environment has a positive effect on perceived trust.*

**H6c.**  *The social environment has a positive effect on self-efficacy for health behaviors*.

**H7a.**  *Consumers’ individual needs for smart packaging have a positive effect on perceived value.*

**H7b.**  *Consumers’ individual needs for smart packaging have a positive effect on perceived trust.*

**H7c.**  *Consumers’ individual needs for smart packaging have a positive effect on self-efficacy for health behaviors.*

#### 3.1.3. Influence of Perceived Value Dimensions on Consumer Purchase Intent and Healthy Eating Behavior

Perceived value is a comprehensive evaluation of the benefits and sacrifices associated with smart packaging by consumers, serving as a critical mediator connecting cognition and behavior [13]. Higher perceived value enhances consumers’ acceptance of food products in smart packaging, thereby increasing purchase intent [52]. Simultaneously, the value recognition of health, safety, and other aspects embedded in perceived value motivates consumers to follow the healthy guidance of smart packaging, leading to healthy eating behaviors [15]. Perceived trust is consumers’ subjective belief in the authenticity of information and reliability of functions of smart packaging [15]. High perceived trust alleviates consumers’ concerns about smart packaging, increasing their purchase intent [53]. Concurrently, trusting the healthy guidance function of smart packaging encourages consumers to be more willing to follow dietary recommendations, fostering healthy eating behaviors [54]. Health behavior self-efficacy is consumers’ judgment of their own ability to achieve healthy eating goals using smart packaging. Stronger self-efficacy enhances consumers’ recognition of the health value of smart packaging, thereby increasing purchase intent [55]. Simultaneously, a sense of efficacy directly motivates consumers to actively practice healthy eating behaviors, translating intention into actual action [56]. Based on this, this paper proposes the following hypotheses:

**H8a.**  *Perceived value has a positive effect on consumers’ intention to purchase smart packaged foods.*

**H8b.**  *Perceived value has a positive effect on consumers’ healthy eating behaviors.*

**H9a.**  *Perceived trust has a positive effect on consumers’ intention to purchase smart packaged foods.*

**H9b.**  *Perceived trust has a positive effect on consumers’ healthy eating behaviors.*

**H10a.**  *Self-efficacy for health behaviors has a positive effect on consumers’ intention to purchase smart packaged foods.*

**H10b.**  *Self-efficacy for health behaviors has a positive effect on consumers’ healthy eating behaviors.*

#### 3.1.4. The Influence of Purchase Intention on Healthy Eating Behaviors

According to the Theory of Planned Behavior, behavioral intention is a direct antecedent of actual behavior; the stronger an individual’s behavioral intention, the higher the probability of implementing the behavior [57]. In the context of smart food packaging, consumers’ intention to purchase smart packaged foods signifies their recognition of its health and safety value. This intention further translates into actual purchasing behavior and, guided by the smart packaging’s health benefits, promotes consumers in developing sustained healthy eating behaviors (e.g., choosing healthy foods, optimizing dietary structure) [58]. Based on this, this study proposes the following hypothesis:

**H11.**  *Consumers’ intention to purchase smart packaged foods has a positive effect on their healthy eating behaviors.*

### 3.2. Theoretical Model

**Figure 2:**
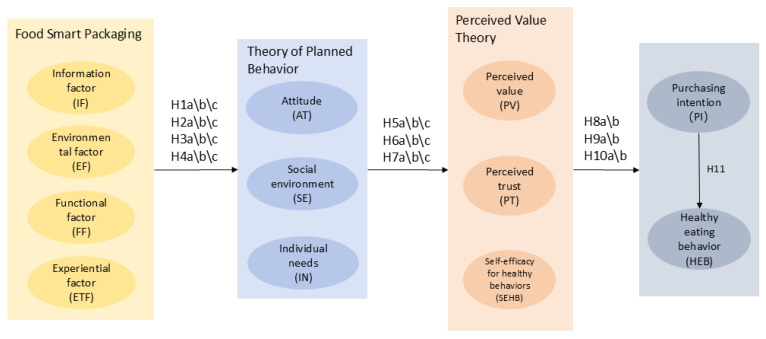
Structural equation model.

Based on the analysis above, we construct the following theoretical model, as shown in Figure 2.

### 3.3. Research Methodology Design

This study aims to investigate the impact mechanism of food smart packaging-related factors on consumers’ purchase intention and healthy eating behaviors, clarifying the causal pathways among the variables. Integrating the exploratory and practical aspects of the research topic, this study adopts a research approach comprising “theoretical deduction—questionnaire survey—empirical testing.” Structural Equation Modeling (SEM) will be employed for hypothesis validation, with Partial Least Squares Structural Equation Modeling (PLS-SEM) selected. PLS-SEM is utilized as the core analytical method for this research primarily for the following reasons: First, this study is a theoretical exploratory study, and as smart food packaging represents a novel technological scenario with an evolving theoretical framework, PLS-SEM is more suitable for theory building and prediction. Second, PLS-SEM can simultaneously handle formative and reflective indicators, aligning with the measurement models of latent variables in this study. Third, PLS-SEM can directly test path relationships among multiple variables, comprehensively addressing the research hypotheses and the demand for mechanism analysis in this study.

This research employed an online sampling method, adhering to the principles of “randomness, representativeness, and feasibility.” The sample covered users from 13 provincial-level administrative divisions in China, including Guangdong, Jiangsu, Zhejiang, Sichuan, and Qinghai provinces. The survey was conducted via internet platforms such as WeChat and Wenjuanxing, randomly selecting online consumers with prior recognition or purchase experience of smart food packaging as survey participants to ensure sample suitability. Concurrently, this study utilized social media convenience sampling, which inevitably introduces self-selection bias. Specifically, the survey sample tends to comprise consumers who are more active on digital platforms, more receptive to online surveys, and more interested in emerging products like smart food packaging. While such a sample can represent the intentions of the majority of consumers, it is difficult to fully represent all food consumer groups, potentially affecting the external validity of the research findings. This can be addressed in future research by expanding sampling channels and supplementing with offline samples. Furthermore, the assessment of sample rationality strictly followed three criteria: 1. Only one questionnaire was accepted per unique IP address; others were eliminated. 2. Questionnaires with repeated selection of the same option for three or more items were deemed invalid and eliminated. 3. Filling times below 180 s were considered perfunctory responses and were excluded.

Moreover, all latent variable measurement indicators were based on established literature, adapted and optimized for the research context. A 7-point Likert scale was utilized for the questionnaire survey to ensure measurement validity. During data analysis, SPSS v.22 and PLS-SEM were employed to progressively evaluate the reliability and validity of the measurement model. Reliability was tested using composite reliability (CR > 0.8), Cronbach’s alpha (Cronbach’s α > 0.7), and factor loadings (>0.7) to ensure internal consistency of the scales. Validity was assessed through content validity, confirmed by literature review, and construct validity, examined via convergent validity (AVE > 0.5) and discriminant validity (Fornell–Larcker criterion and HTMT) to ensure dimensional distinctiveness and guarantee the scientific rigor of the measurement model. To address potential common method bias (CMB) in cross-sectional survey research, controls were implemented at three levels: at the research design level, anonymous surveys, randomized item order, and distinct phrasing for predictive and outcome variables were employed to mitigate bias; at the data collection level, valid questionnaires were screened, and multi-scenario surveys were conducted to ensure data authenticity; at the statistical testing level, Harman’s single-factor test revealed that the variance explained by the first common factor was less than 40%, indicating that CMB was within an acceptable range. 

#### 3.3.1. Sample Selection and Data Collection

The target respondents for this study are consumers with experience purchasing food and who are familiar with or have used smart food packaging (e.g., digital labels, RFID-traced packaging, freshness indicator packaging). The sample encompasses diverse age groups, genders, educational backgrounds, and income levels to ensure representativeness. Data collection was conducted via online questionnaires distributed through platforms like Wenjuanxing and social media, over a period from March 2025 to January 2026. In addition to basic demographic information, all construct items were measured using a 7-point Likert scale, ranging from 1 (Strongly Disagree) to 7 (Strongly Agree). The selection of a 7-point Likert scale over a 5-point scale was based on two primary considerations. First, consumer perceptions of smart food packaging often involve nuanced differences. A 5-point scale, with its broader categories of “Disagree,” “Neutral,” and “Agree,” may struggle to capture these subtle perceptual variations. The 7-point scale, by incorporating intermediate levels such as “Somewhat Disagree” and “Somewhat Agree,” allows for a more precise and granular characterization of consumer evaluations across different dimensions of smart food packaging, thereby enhancing measurement accuracy. Second, from the perspective of structural equation modeling (SEM) suitability, the measurement outcomes from a 7-point scale approximate continuous variables more closely, better satisfying the interval measurement assumption of SEM. This improves the precision and reliability of model estimation and mitigates potential model fit biases arising from the coarser, more discrete nature of a 5-point scale, thereby ensuring the rigor of subsequent data analysis and hypothesis testing.

The data collection adhered to the principles of “clarity, objectivity, and logic” and was structured into three parts. The first part comprised the survey introduction and informed consent, outlining the research objectives, confidentiality protocols, and questionnaire completion guidelines. The second part gathered demographic information, including gender, age, education level, monthly income, and food purchase frequency. The third part contained the core measurement items, covering 12 latent variables: consumer attitudes toward smart food packaging, social influences, individual needs, environmental factors of smart packaging, functional factors, experiential factors, informational factors, perceived value, perceived trust, health behavior self-efficacy, purchase intention, and healthy eating behavior. A total of 622 questionnaires were distributed, with 602 returned. After screening and exclusion of invalid responses—such as duplicates, incomplete entries, logical inconsistencies, excessive use of the same option, or response times under 180 s—566 valid questionnaires were obtained, yielding a valid response rate of 94%.

**Figure 3:**
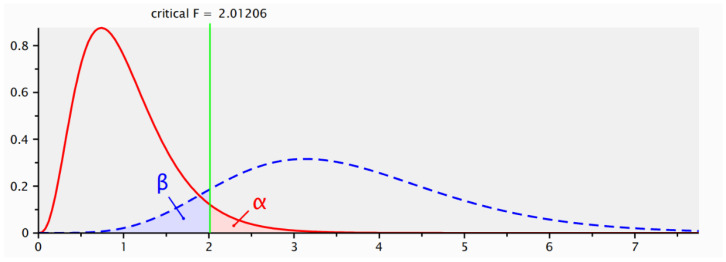
G*power analysis graph.

As depicted in Figure 3, the statistical power analysis results from G*Power 3.1 were generated for a linear multiple regression model, specifically focusing on the R^2^ deviation of a fixed model. The input parameters included an effect size (f^2^) set at 0.15, a significance level (α error probability) of 0.05, and a desired statistical power (1-β error probability) of 0.90. This power level indicates a 90% probability of correctly rejecting the null hypothesis when it is false. Under these conditions, G*Power calculated a total required sample size of 136. This sample size ensures that the study possesses adequate statistical power to detect the hypothesized effects within the model, given the specified effect size and significance level. The actual statistical power achieved was 0.9022099, slightly exceeding the target of 0.90, confirming that this sample size meets the research design requirements and supports the reliability of the study’s findings. Therefore, the 566 valid responses collected from consumers who had encountered or purchased smart packaging foods are deemed sufficient and appropriate, particularly given the 36 questions included in the questionnaire, which resulted in 566 valid answers. This substantiates the validity and reasonableness of the sample size.

#### 3.3.2. Informed Consent

This study received ethical approval from the Ethics Review Committee of the School of Fine Arts, Nanjing Normal University (NO.NNU SFA-E-2024-012). Prior to commencing the questionnaire survey, an online informed consent form is automatically presented. Participants proceed to the survey page only after providing verbal consent and selecting the agreement option; refusal immediately terminates the survey. Considering that minors lack full legal capacity, this survey does not recruit minors. Participants must be 18 years of age or older to ensure they possess sufficient cognitive and comprehension abilities to accurately understand the questionnaire content and provide valid feedback. Participants are required to have actually purchased or encountered smart food packaging to guarantee they have direct experience with the domain of smart food packaging relevant to the study, thereby enabling the provision of authentic and reliable survey data.

Respondents completed the questionnaire via the Wenjuanxing online survey platform. The primary channels for survey distribution were social media platforms such as WeChat, utilizing methods like forwarding through WeChat Moments and relevant comment sections on Rednote to guide users with experience in purchasing or interacting with smart food packaging to participate in the survey. In consideration of practical survey conditions and the feasibility of online research, this study adopted an online convenience sampling method. The sampling regions primarily covered first-tier, new first-tier, second-tier, and third-tier cities in China, with a focus on the East China, South China, and North China regions where smart food packaging is relatively concentrated, while also including central and western regions to expand geographical coverage as much as possible.

#### 3.3.3. Measurement Questionnaire Design

The scales for all latent variables in this study were adapted from existing, established literature. Integrating the characteristics of smart food packaging, the TPB theoretical model, and the theory of perceived value, the item statements were optimized to ensure the applicability and validity of the scales. All items utilized a 7-point Likert scale. The specific scale designs are as follows:

Core characteristics of smart packaging were designed across four dimensions: functional factors, experiential factors, environmental factors, and safety factors. Items (IF1–IF3) were designed by referencing content on information factors of smart food packaging from Butt, Huang, Pokhrel, and Yang [2,3,4,5]. Items (EF1–EF3) were designed by referencing content on environmental factors of food packaging from Lindh and Steenis [29,30]. Items (FF1–FF3) were designed by referencing content on functional factors of smart food packaging from Aday [28]. Items (ETF1–ETF3) were designed by referencing content on experiential factors of food packaging from Aday and Steenis [28,29].

The consumer attitude scale (AT1–AT3) was designed with reference to the research of Ajzen and Gigauri [12,47]. The social environment scale (SE1–SE3) was designed with reference to the Planned Behavior Theory content of Ajzen and Li [12,24]. The consumer individual need scale (IN1–IN3) was designed with reference to Chen’s [14] work on consumer perceived behavioral control. The consumer perceived value scale (PV1–PV3) was designed with reference to relevant content from Zeithaml, Ellen, Sheth, and Johnson [13,39,40,41]. The consumer perceived trust scale (PT1–PT3) was designed with reference to relevant content from Rafsabdjani and Young [15,53]. The health behavior self-efficacy scale (SEHB1–SEHB3) was designed with reference to relevant content from Zafar and Chang [55,56].

The consumer purchase intention scale (PI1–PI3) was designed with reference to relevant content from Rafsandjani and Armitage [53,57]. The healthy eating behavior scale (HEB1–HEB3) was designed with reference to relevant content from Singh and Zafar [56,58]. Based on the above analysis, the items for questionnaire design are shown in Table 1.

## 4. Data Analysis

### 4.1. Sample Statistical Analysis

A total of 566 valid samples were collected in this survey, indicating a sufficient sample size that meets the criteria for PLS-SEM analysis (Table 2). From a demographic perspective, the gender distribution is balanced, with the majority of participants falling within the 25–54 age range. The occupational distribution covers various mainstream employment types, and the educational attainment is predominantly at the undergraduate level or higher. The income distribution is primarily composed of low to middle-income groups. Overall, the distribution is reasonable and highly consistent with the demographic structure of urban food consumers in China, thereby comprehensively reflecting the cognition and behavioral characteristics of different groups towards smart packaging. Regarding contact with and purchase of smart packaging, over 70% of respondents have had relevant exposure experiences. The purchase frequency exhibits a normal distribution with a higher concentration in the middle range and lower frequencies at the extremes, which aligns with the current market penetration stage of smart packaging. The respondents possess a foundation of real-world experience, and the data shows no significant extreme deviations.

In summary, the sample distribution across dimensions such as gender, age, occupation, education, and income is balanced, with no apparent sampling bias. The core demographic aligns closely with the target consumer group for smart packaging, demonstrating strong typicality and representativeness. The sample structure is scientifically sound and the data is authentic and valid, adequately supporting subsequent model testing and mechanism analysis, thus providing a robust data foundation for the reliability and generalizability of the research findings.

**Table 2:** Demographic profile of sample (*n* = 566).

Sample	Category	Number	Percentage %
Gender	Male	310	54.77
	Female	256	45.23
Age	18–24	100	17.67
	25–34	115	20.32
	35–44	160	28.27
	45–54	113	19.96
	Over 55	78	13.78
Occupation	Student	70	12.37
	Freelance	85	15.02
	Self-employed	120	21.20
	Public officials or public institutions	135	23.85
	Foreign-funded enterprise	96	16.96
	Private enterprise	60	10.60
Average monthly disposable income	Below 1000 yuan	65	11.48
	1001–3000 yuan	198	35.00
	3001–5000 yuan	152	26.86
	5001–8000 yuan	72	12.72
	Above 8001 yuan	79	13.96
Have you ever come across food products with intelligent packaging?	Rare contact	72	9.84
	Occasional contact	216	29.51
	Frequent contact	249	34.02
	Daily contact	29	3.96
The frequency of purchasing smart-packaged food	Two to three times a week	61	10.7
	Two to three times a month	168	29.7
	Two to three times a quarter	198	35
	Two to three times in half a year	139	24.6

### 4.2. Reliability and Validity Analysis

Reliability reflects the stability and consistency of a measurement tool [59]. This study employed Cronbach’s alpha coefficient to assess the reliability of the scales, using SPSS 22.0 software for analysis. Concurrently, the variance inflation factor (VIF) was utilized to test for multicollinearity [60]. The results indicate that all measurement variables have Cronbach’s alpha values exceeding 0.874, which is above the threshold of 0.7 [61]. The alpha values after removing any item did not exceed the current results, and the correlations between removed items and the total score were all greater than 0.5. This suggests that the questionnaire items are well-designed, require no deletion, and the scales possess good reliability. In the multicollinearity test, all variables had VIF values below 2.531, significantly lower than the critical value of 5 [62], and consistent with the ideal condition of VIF < 3.3 [63]. This indicates low correlation among the independent variables and no apparent multicollinearity issues. Therefore, the scales used in this study exhibit good reliability, and the data is dependable, free from multicollinearity interference, and suitable for subsequent model construction and mechanism analysis (Table 3).

**Table 3:** Reliability analysis results (*n* = 566).

Dimension	Items	Collinearity Statistics (VIF)	Corrected Item-to-Total Correlation	Cronbach’s α if Item Deleted	Cronbach’s α
IF	IF1	2.256	0.746	0.831	0.874
	IF2	2.396	0.763	0.816	
	IF3	2.392	0.762	0.817	
EF	EF1	2.354	0.755	0.808	0.868
	EF2	2.100	0.723	0.837	
	EF3	2.434	0.766	0.798	
FF	FF1	2.306	0.744	0.826	0.871
	FF2	2.273	0.751	0.819	
	FF3	2.223	0.763	0.809	
ETF	ETF1	2.340	0.757	0.810	0.869
	ETF2	2.264	0.747	0.819	
	ETF3	2.259	0.746	0.819	
AT	AT1	2.128	0.728	0.808	0.859
	AT2	2.139	0.729	0.807	
	AT3	2.241	0.744	0.793	
SE	SE1	2.142	0.730	0.795	0.855
	SE2	2.099	0.724	0.800	
	SE3	2.127	0.728	0.796	
IN	IN1	2.170	0.732	0.797	0.857
	IN2	2.022	0.711	0.817	
	IN3	2.258	0.746	0.784	
PV	PV1	2.291	0.750	0.816	0.869
	PV2	2.232	0.743	0.823	
	PV3	2.346	0.757	0.809	
PT	PT1	2.322	0.754	0.819	0.872
	PT2	2.438	0.768	0.806	
	PT3	2.216	0.740	0.831	
SEHB	SEHB1	2.531	0.778	0.804	0.874
	SEHB2	2.246	0.744	0.835	
	SEHB3	2.320	0.752	0.827	
PI	PI1	2.088	0.721	0.806	0.856
	PI2	2.233	0.743	0.786	
	PI3	2.101	0.723	0.804	
HEB	HEB1	2.286	0.750	0.821	0.871
	HEB2	2.376	0.761	0.811	
	HEB3	2.267	0.747	0.823	

Common method bias (CMB) was tested using the classic Harman’s Single-Factor Test. The core logic of this method is that if significant common method bias exists, all variables will aggregate into a single factor in an unrotated exploratory factor analysis (EFA), or the variance explained by this single factor will exceed a specific critical threshold [64]. Podsakoff and Organ proposed a clear criterion: if the variance explained by the single factor obtained through unrotated EFA does not exceed 50%, it indicates that the common method bias is within a controllable range [65].

This study employed SPSS software and utilized Harman’s Single-Factor Test to examine common method bias. The results, presented in Table 4, show that all items automatically grouped into three factors with eigenvalues greater than 1, explaining a cumulative variance of 50.627%. Specifically, the variance explained by the first factor before rotation was 39.633%, which did not reach the critical standard of 50%, and no single factor explained the majority of the variation. The aforementioned results indicate that the common method bias in this study has been effectively controlled and will not significantly impact the reliability of the research findings.

At the same time, the CMB was tested using the potential common method factor. According to the research of Williams et al., a common method factor was included in the model [66,67]. As shown in Table 5, the results indicated that the average market value of the indicators was explained by 0.795664, while the average method variance was 0.003481. The ratio of substantive difference to method difference was approximately 228:1, and the method variation accounted for only 0.44% of the total variation, which was far below the critical standard of 10%, indicating that the common method bias did not pose a serious threat to the results of this study [68].

This study employed the Kaiser-Meyer-Olkin (KMO) and Bartlett’s tests of sphericity for exploratory factor analysis (EFA) to ascertain the correlational appropriateness of variables for factor extraction [69]. As presented in Table 6, the KMO values for each variable ranged between 0.733 and 0.742, all exceeding the critical threshold of 0.5. Furthermore, the significance values for Bartlett’s test of sphericity were consistently below 0.05 and approached zero, indicating that the data met the prerequisites for factor analysis [70]. Principal component analysis revealed that only one common factor with an eigenvalue greater than 1 was extracted for each variable, with cumulative variance contribution rates exceeding 50%. All items demonstrated common factor variances greater than 0.5 and factor loadings exceeding 0.6, which aligns with the reference standards from prior research [71], suggesting the data exhibits good unidimensionality.

Through confirmatory factor analysis (CFA), this study found that the factor loadings for all scale items were above 0.5. This indicates that all items comprising each variable consistently explain that variable. This consistency not only signifies that the items effectively capture the essence of the variable but also demonstrates the scale’s high stability and reliability in measuring that variable [72]. Based on the item factor loadings, composite reliability (CR) and average variance extracted (AVE) were further calculated. According to established criteria, CR values should not be below 0.7, and the minimum standard for AVE is 0.5 [73]. As shown in Table 7, the factor loadings for all items in this study exceeded 0.802, CR values surpassed 0.855, and the square roots of AVE were all greater than 0.663, indicating good convergent validity for the relevant variables.

The Heterotrait–Monotrait (HTMT) ratio serves as a critical indicator for rigorously assessing the discriminant validity of constructs within structural equation modeling (SEM). Typically, an HTMT value below 0.85 signifies good discriminant validity between variables [74]. This test is crucial for mitigating issues arising from high inter-construct correlations, which can lead to multicollinearity and consequently affect model efficacy. The results of this study (refer to Table 8) show that all HTMT values ranged between 0.656 and 0.845, all satisfying the critical threshold of 0.850. Consequently, the model demonstrates sufficient discriminant validity, and the empirical findings are robust and reliable.

Meanwhile, 5000 bootstrap resamples were used to calculate the 90% percentile confidence intervals (5.0–95.0%) of the paired correlation coefficients of each variable. As shown in Table 9, the confidence intervals of all variable pairs are positive, do not include 0, and have a compact width with stable upper and lower limits. The two mean estimates are highly consistent, and the bias is almost all 0, with only a few pairs being ±0.001, and there is no systematic bias. According to the HTMT discriminant validity standard [74], the confidence intervals do not cross 0, are narrow, the estimates converge, and there is no abnormal deviation, indicating that the parameter estimates are robust, statistically significant, and reliable, meeting the requirements of the confidence intervals for structural equation models.

Discriminant validity examines the degree of mutual distinction between different latent variables. Adhering to the Fornell–Larcker criterion, discriminant validity is considered adequate when the correlation coefficients between variables do not exceed 0.9 [75]. Table 10 indicates that the square roots of AVE for each latent variable are greater than their respective correlation coefficients with other latent variables. The maximum correlation coefficient observed was 0.890, which did not surpass the critical value of 0.9. Furthermore, the diagonal values (square roots of AVE) are greater than the off-diagonal values within their respective rows and columns. These results suggest that while variables are significantly correlated, their boundaries are clearly demarcated, and distinction is good, indicating the measurement model possesses desirable discriminant validity.

### 4.3. Model Fit Test

Table 11 reports the coefficient of determination (R^2^), adjusted R^2^, predictive relevance (Q^2^), and goodness of fit (GOF) for each dimension. R^2^ reflects the explanatory power of the model for the dependent variable; generally, an R^2^ > 0.6 is considered ideal. A Q^2^ > 0 indicates predictive relevance, and a GOF value higher than 0.35 signifies excellent overall model fit [76,77]. As depicted in Table 9, the R^2^ values for Individual Needs (IN), Social Environment (SE), Healthy Eating Behavior (HEB), Attitudes (AT), Perceived Value (PV), Perceived Trust (PT), Consumer Intention (PI), and Self-Efficacy for Healthy Behaviors (SEHB) are 0.839, 0.830, 0.828, 0.843, 0.830, 0.838, 0.832, and 0.832, respectively, all exceeding the ideal standard of 0.6, indicating excellent explanatory power. All dimensions have Q^2^ values greater than 0, suggesting good predictive capability of the model. The overall GOF is 0.753, which is above the critical threshold of 0.35 for excellent fit, indicating robust overall model fit.

The SRMR is a commonly used metric for assessing model fit in structural equation modeling, with lower values indicating better fit, and a value below 0.08 denoting good fit [78]. Both d-ULS and d-G values are judged as good fit when below 0.95 [79,80], while NFI has a threshold greater than 0.8 for a relatively good fit [81]. As shown in Table 12, this study’s SRMR is 0.028, below the 0.08 critical value; d-ULS is 0.518 and d-G is 0.832, both less than 0.95; and NFI is 0.882, exceeding the 0.8 standard. All fit indices meet their respective criteria, indicating an ideal overall model fit

### 4.4. Path Hypothesis Analysis

This study employed PLS-SEM combined with bootstrapping of 5000 resamples to test the research hypotheses, with a significance threshold of *p* < 0.05. The results indicated that all 28 path coefficients were significant at the *p* < 0.001 level, as detailed in Table 13, providing empirical support for all research hypotheses.

Regarding the influence of smart packaging dimensions on Theory of Planned Behavior (TPB) constructs, the four factors of information, environmental friendliness, functionality, and experience all significantly and positively drove attitude, social environment, and individual needs. Specifically, the path coefficients for the information factor were β = 0.221, β = 0.272, and β = 0.241; for the environmental friendliness factor, they were β = 0.235, β = 0.224, and β = 0.247; for the functionality factor, they were β = 0.282, β = 0.228, and β = 0.204; and for the experience factor, they were β = 0.227, β = 0.235, and β = 0.271. The mediating effect of TPB constructs on perceived value systems was also significant: attitude had path coefficients of β = 0.323, β = 0.284, and β = 0.247 for perceived value, perceived trust, and self-efficacy, respectively; the social environment had coefficients of β = 0.223, β = 0.360, and β = 0.292; and individual needs exhibited the strongest driving effect, with coefficients of β = 0.407, β = 0.314, and β = 0.414.

The perceived value construct exerted a significant positive influence on behavioral outcomes: the path coefficients for perceived value to purchase intention and healthy eating were β = 0.303 and β = 0.201, respectively; for perceived trust, they were β = 0.344 and β = 0.316; and for self-efficacy, they were β = 0.306 and β = 0.203. Concurrently, the path coefficient from purchase intention to healthy eating behavior was β = 0.236. Meanwhile, in the multicollinearity test, the VIF values of all paths were below 4.291, which is lower than the critical value of 5 [62]. This indicates a relatively low correlation and no interference from multicollinearity. These findings comprehensively validated the effectiveness and rationality of the chained mediation model, extending from smart packaging perceptions to TPB constructs, then to perceived value mediation, and ultimately influencing purchase intention and healthy eating behavior.

**Figure 4:**
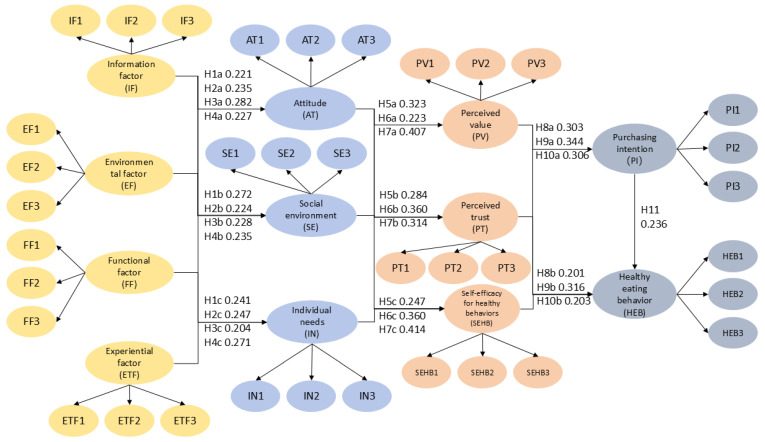
Hypothesis test results.

The results of hypothesis testing are shown in the above table, as shown in Figure 4.

### 4.5. Importance and Performance Analysis of IPMA

IPMA is an analytical method for comprehensively evaluating the strategic importance and performance of each construct [82]. The horizontal axis represents relative importance (total effect on the target construct), while the vertical axis presents performance (measured on a standardized scale from 0 to 100). To examine differences, we conducted a specific generational IPMA test for healthy eating behavior (HEB) and compared the coordinate patterns across key value dimensions.

**Figure 5:**
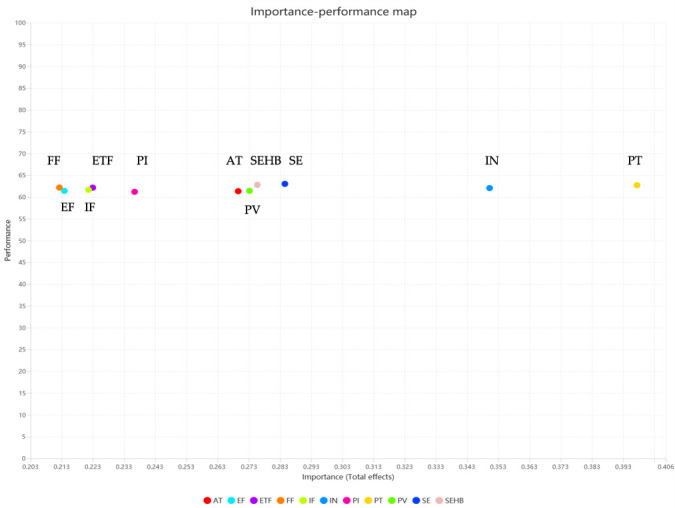
Importance–Performance Analysis Chart.

Figure 5 displays the Importance-Performance map of influencing factors for consumer behavior related to smart food packaging. In terms of importance (total effect), perceived trust (PT) holds the highest importance (approximately 0.400), serving as the core variable influencing consumers’ healthy eating behavior. This is followed by individual needs (IN, approximately 0.350) and the social environment (SE, approximately 0.283); self-efficacy (SEHB, approximately 0.280), perceived value (PV, approximately 0.273), attitude (AT, approximately 0.273), consumer intention (PI, approximately 0.236), experience (ETF, approximately 0.223), information (IF, approximately 0.220), environmental friendliness factor (EF, approximately 0.213), and functionality factor (FF, approximately 0.210) show successively decreasing importance. This suggests that consumers, in the process of accepting smart food packaging and forming behaviors, prioritize core psychological variables such as trust-building, intrinsic needs, and social norms, rather than basic peripheral factors like functionality and environmental attributes.

In terms of performance, the overall performance scores of the variables fall within the 60–64 score range. Functionality (FF, approximately 63 points), the social environment (SE, approximately 63 points), and self-efficacy (SEHB, approximately 63 points) demonstrate the most prominent performance, categorized as “strengths” in consumer perceived experience. Perceived trust (PT, approximately 62 points), experience (ETF, approximately 62 points), individual needs (IN, approximately 62 points), information (IF, approximately 61 points), perceived value (PV, approximately 61 points), the environmental friendliness factor (EF, approximately 61 points), attitude (AT, approximately 60 points), and consumer intention (PI, approximately 60 points) exhibit moderate performance levels, indicating stable overall performance.

Analysis of the synergistic relationship between importance and performance indicates that perceived trust (PT), despite holding the highest importance, exhibits only an upper-medium performance, categorizing it as a “key improvement area.” This identifies PT as a critical breakthrough point for optimizing the food smart packaging experience and enhancing consumer trust perception. Factors such as individual need (IN), social environment (SE), functionality (FF), and self-efficacy (SEHB) not only hold high importance but also demonstrate stable performance, thus belonging to the “advantage zone.” These factors currently represent the core strengths supporting consumers’ purchase intentions and healthy eating behaviors. Experience, information, attitude, perceived value, consumer intention, and environmental factors all fall within the “maintenance zone,” with both importance and performance at moderate levels. Continued effort should focus on consolidating existing performance while moderately optimizing these areas to enhance overall competitiveness.

Overall, the formation of consumer behavior related to food smart packaging is highly dependent on a dual logic of “core psychological drivers and fundamental functional support.” Subsequent optimization efforts should primarily concentrate on elevating the perceptual experience of perceived trust, while concurrently consolidating the leading positions of advantageous factors like individual need, social environment, and functionality. By balancing the synergy between importance and performance, the conversion of consumer purchase intentions into healthy eating behaviors can be comprehensively promoted. From the perspective of IPMA numerical characteristics, the performance scores of all variables are concentrated within a relatively narrow range of 60–64/100, indicating that the performance differences between variables are not significant. This also presents a certain limitation for the IPMA analysis in this study. To address the issue of a narrow performance score range, future research could further optimize the evaluation indicator system to broaden the performance score differences among variables, thereby enhancing the discriminative power and scientific rigor of the analysis results.

## 5. Discussion

This study constructed a chained mediating model: “Perceived Factors of Food Smart Packaging → Core Constructs of TPB → Dimensions of Perceived Value → Purchase Intention → Healthy Eating Behavior.” The model and research hypotheses were validated using PLS-SEM and IPMA. The results demonstrated that all research hypotheses were empirically supported and exhibited good validity and rationality. This research not only enriches the existing literature on food smart packaging and consumer behavior but also provides practical and actionable guidance for the optimization and upgrading of food smart packaging and the promotion of healthy consumption. This is further corroborated by comparing the empirical results with existing studies.

In terms of specific analysis, the four perceived factors of food smart packaging—information, environmental friendliness, functionality, and experience—all exert a significant positive influence on the three core constructs of the TPB: attitude, social environment, and individual need. This finding aligns with conclusions from other scholars regarding how perceived attributes of smart packaging can trigger changes in consumer psychological cognition [27,83]. Existing literature widely posits that the functional utility and information transparency of smart packaging are the core external stimuli influencing consumer attitudes and behavioral intentions [1,15,27]. For instance, Htun et al. found that the functional reliability of smart packaging can significantly enhance consumers’ positive attitudes toward products [11,50], and Spada et al. confirmed that the way information is presented on smart packaging affects consumers’ perceptions of social norms [84,85]. The conclusions of this study regarding the influence of functional and informational factors align with these previous findings, further validating the driving role of external perceptual factors on consumer psychological cognition. This study found that experiential factors have the strongest drive for personal needs (β = 0.271, *p* < 0.001), highlighting the crucial role of smart packaging’s operational convenience and user-friendliness in enhancing consumers’ sense of control over usage, which aligns with consumers’ practical focus on user experience in daily consumption. This result complements the deficiencies in existing research, which has predominantly focused on the influence of functional and informational factors [1,15,27] with less attention paid to experiential factors. Existing studies have only mentioned that the interactive experience of smart packaging might influence consumers’ willingness to use it [86], but have not clearly defined its specific driving strength for personal needs. This study, through empirical data, has clarified the core role of experiential factors, aligning with consumers’ practical emphasis on user experience in daily consumption. Furthermore, environmental factors also have a relatively strong influence on personal needs (β = 0.247, *p* < 0.001). This is consistent with the research context of the rising green consumption concept and aligns with Li et al.’s conclusion that “consumers’ green consumption needs positively respond to the environmental attributes of smart packaging” [24,47], reflecting that under the green consumption ideology, the environmental attributes of smart packaging can effectively meet consumers’ sustainable consumption needs. The path coefficients of informational factors on attitudes, social environment, and personal needs are β = 0.221, β = 0.272, and β = 0.241, respectively. The path coefficients of functional factors on these three are β = 0.282, β = 0.228, and β = 0.204, respectively. Among these, functional factors have the strongest drive for attitudes, while informational factors have a more prominent influence on the social environment. This nuanced difference supplements the generalized statements in existing research, which often broadly states that informational and functional factors have positive influences without clearly delineating their differential impacts on various TPB constructs. The empirical results of this study further refine the characteristics of the role of external perceptual factors, indicating that food information transparency, functional reliability, and operational convenience are key to consumers forming positive attitudes and being influenced by social norms. These four factors constitute a synergistic system of external stimuli, laying the foundation for subsequent value judgments and behavioral intention formation, which corroborates Natalia et al.’s viewpoint that “multiple perceptual factors of smart packaging synergistically influence consumer psychological cognition” [84,85,87].

Secondly, the mediating effect of the core constructs of TPB on perceived value revealed that individual needs most strongly drove perceived value, perceived trust, and self-efficacy (β values were 0.407, 0.314, and 0.414, respectively, all *p* < 0.001). This confirms that consumers’ intrinsic needs for smart packaging (e.g., safety, convenience, health) are the core drivers of their value judgments and trust perceptions, aligning with the core tenet of the Theory of Planned Behavior where perceived behavioral control (operationalized here as individual needs) influences individual value appraisals and behavioral confidence [10,88]. This aligns strongly with the theoretical framework proposed by Ajzen, which posits that “perceived behavioral control indirectly influences behavior by affecting an individual’s psychological evaluation [12].” Furthermore, this result resonates with existing empirical studies. For instance, Davidescu et al. found that consumers’ demand for smart packaging positively influences their perceived value [1,84]. Zhou also confirmed that stronger perceived behavioral control leads to higher consumer trust in smart packaging [89]. This study further expands upon these findings by explicitly quantifying the specific driving strength of personal needs across the dimensions of perceived value, perceived trust, and self-efficacy, thereby refining the mediating pathways. The study revealed that the social environment exerts the strongest influence on perceived trust (β = 0.360, *p* < 0.001), with path coefficients for perceived value and self-efficacy being β = 0.223 and β = 0.292, respectively. This highlights the impact of social consensus and group demonstration effects on smart packaging, effectively enhancing consumer trust in the packaging’s information and functionalities. This finding is consistent with Liu’s research, which concluded that “perceived social norms positively influence consumers’ trust in smart products [90].” Additionally, attitudes consistently exert a stable positive influence on all three dimensions of perceived value (path coefficients of β = 0.323, β = 0.284, and β = 0.247, respectively), serving as a critical link in the transmission from psychological cognition to value judgment. This is in congruence with Ajzen’s view within the Theory of Planned Behavior that “attitude is the core mediator connecting external stimuli to behavioral intentions [12],” and it substantiates the conclusion from existing research that “positive consumer attitudes toward smart packaging enhance their perceived value and trust [1,84]”.

Concurrently, the influence of the perceived value dimension on behavioral outcomes indicates that perceived trust is the strongest driver of healthy eating behavior (β = 0.316, *p* < 0.001), followed by its influence on purchase intention (β = 0.344, *p* < 0.001). This suggests that consumer trust in smart packaging is central to forming healthy eating behaviors, and only by trusting the packaging’s information and functionalities will consumers adhere to healthy guidance [91,92]. This finding aligns strongly with existing research, such as Spada’s study, which found that consumer trust in smart packaging is a critical factor influencing their healthy consumption behavior, with perceived trust positively driving consumers’ purchase intentions and health-related behaviors [84]. This study further quantifies the driving strength of perceived trust through empirical data and clarifies the differential impact on purchase intention and healthy eating behavior, addressing the quantitative analysis limitations in existing research. Perceived value and self-efficacy both exert significant positive influences on purchase intention and healthy eating behavior, with path coefficients for perceived value being β = 0.303 and β = 0.201, and for self-efficacy being β = 0.306 and β = 0.203, respectively. Self-efficacy’s influence on purchase intention is slightly higher than perceived value, underscoring the importance of consumers’ self-perception of capability in their purchasing decisions. This is consistent with Bandura’s theory of self-efficacy, which states that “an individual’s sense of self-efficacy influences their behavioral decisions and execution [93].” It also addresses a gap in existing research concerning the differential influence of perceived value and self-efficacy. While existing studies often mention their positive impact simultaneously [94], they have not explicitly detailed the subtle differences in their influence strengths. The empirical results of this study fill this void. The significant positive influence of purchase intention on healthy eating behavior (β = 0.236, *p* < 0.001) further validates the TPB’s view that behavioral intention is a direct antecedent of actual behavior, indicating that purchase intention can be effectively translated into healthy eating behavior and extending the applicability of the TPB to the consumption scenario of smart food packaging.

Integrating the results from the IPMA, perceived trust (PT) exhibits the highest importance, functioning as a core determinant influencing healthy eating behavior. However, its performance is at a medium-to-high level, placing it in the “key improvement area.” This finding aligns with the path analysis conclusions, suggesting that enhancing consumers’ perceived trust should be a primary focus for future efforts. This outcome is consistent with the findings of Spada et al., who concluded that “perceived trust is a core driver of consumer behavior for smart packaging, yet current consumer trust in smart packaging still has room for improvement” [1,84]. It also corroborates the viewpoint in existing research that “a lack of trust is a critical issue limiting the market penetration of smart packaging” [15], reinforcing the recommendation to prioritize improving consumer perceived trust. Factors such as individual needs (IN), social environment (SE), functionality (FF), and self-efficacy (SEHB) demonstrate high importance and stable performance, categorizing them within the “strength area.” These factors serve as the core driving forces supporting consumer behavior and should be further reinforced. This aligns with the conclusion from existing studies that “individual needs and functional factors are key drivers of smart packaging consumption” [50], further validating the central value of these elements. Experience, information, attitude, perceived value, purchase intention, and environmental factors all fall within the “maintain area,” exhibiting medium levels of both importance and performance, necessitating moderate optimization. Notably, environmental factors rank lowest in the overall analysis, consistent with their relatively moderate influence observed in the path analysis (beta coefficients of 0.235 for attitude, 0.224 for social environment, and 0.247 for individual needs). This result echoes some existing research—Htun et al. found that consumers’ current focus for smart packaging remains on functional utility and information transparency, with relatively lower attention paid to environmental attributes [1,50]. Through the dual validation of IPMA and path analysis, this study further confirms this conclusion, reflecting that current consumer attention towards smart packaging is concentrated on core functionalities and psychological values rather than solely on environmental attributes, providing clear directional guidance for subsequent research and practical optimization.

Based on the aforementioned analyses, this study draws the following core conclusions:

First, experiential factors are the core elements with the strongest driving effect on individual needs (perceived behavioral control) among the perceived dimensions of smart packaging (β = 0.271, *p* < 0.001), with a significantly higher impact strength than informational factors (β = 0.241), environmental factors (β = 0.247), and functional factors (β = 0.204). This finding effectively complements existing research that often emphasizes the core role of functional factors, clearly revealing the critical value of “operational experience convenience” in lowering consumer usage barriers and enhancing their sense of behavioral control, thereby challenging the traditional perception that “function precedes experience.” Second, individual needs (perceived behavioral control) exhibit the most significant driving effects on perceived value, perceived trust, and self-efficacy (β values are 0.407, 0.314, and 0.414, respectively), indicating that consumers’ intrinsic needs for smart packaging serve as the core logical starting point for their value judgments and behavioral decisions. This conclusion further refines the mechanism of “perceived behavioral control” within the context of smart packaging consumption as per the Theory of Planned Behavior, clarifying the ambiguity in existing research regarding the differential impact strengths of the three constructs of the Theory of Planned Behavior. Third, perceived trust has a significantly stronger driving effect on healthy eating behavior (β = 0.316, *p* < 0.001) compared to perceived value (β = 0.201) and self-efficacy β = 0.203), confirming that “trust is the core link for behavioral transformation.” This finding fills a gap in existing research concerning the priority of value judgment variables, clearly establishing perceived trust as a central hub in the “value judgment–behavioral outcome” pathway. Fourth, IPMA results indicate that perceived trust (PT) is the variable with the highest importance, yet its performance level is only above average. Concurrently, the importance and performance of environmental factors are significantly lower than other perceived variables. This conclusion clearly reveals the current characteristics of the smart packaging consumption market, namely that “core trust needs are not fully met” and “environmental attributes are not the primary consumer concern.” It provides precise targets for future smart packaging optimization directions, presenting a distinctly differentiated finding from existing research that emphasizes the importance of environmental and functional aspects.

### 5.1. Theoretical Contributions

Synthesizing the discussion with the empirical data from this study, the theoretical contributions primarily lie in supplementing and refining existing research. This is manifested in three key areas:

Firstly, it addresses the deficiency in existing research that isolates single factors or theories. Much of the prior work has failed to construct a comprehensive analytical framework encompassing multiple perceptual factors, psychological constructs, and value variables in relation to behavioral outcomes. Consequently, the intrinsic mechanisms through which smart packaging influences consumers’ healthy eating behaviors have not been fully elucidated. Based on empirical data, this study systematically integrates four core factors with the TPB and Perceived Value Theory. It constructs and validates a complete chained mediation model, clarifying the transmission pathways between variables. This work, to some extent, fills the research gap concerning the mechanisms of multivariate synergistic effects, thereby rendering related research conclusions more systematic and comprehensive.

Secondly, it refines the application details of the TPB in the context of smart packaging. Existing studies have often defined the operationalization of perceived behavioral control generically as a “sense of control over behavior,” without specific optimization for smart packaging scenarios. Furthermore, the transmission pathways of TPB’s core constructs to value judgment variables have not been explicitly detailed. This study clarifies the specific transmission pathways of TPB’s core constructs to perceived value, perceived trust, and self-efficacy. It refines the application methods of the TPB in emerging technology consumption scenarios, thereby expanding its explanatory boundaries within a certain scope and mitigating the issue of a weak link between theoretical application and specific contexts.

Finally, it compensates for the limitation in previous research that prioritized path verification over priority judgment. Much of the existing research has focused on verifying significant relationships between variables without clearly identifying the importance and performance differences of each variable. This has led to an inadequate understanding of core driving factors and optimization priorities. This study, integrating IPMA with path verification results and based on empirical data, clearly delineates the differences in influence intensity and optimization priority among variables. This provides a more targeted analytical perspective and research framework for subsequent related studies, thereby enriching the research methodologies in relevant fields.

### 5.2. Practical Value

Drawing upon the empirical conclusions, research findings, and IPMA results from this study, and considering the characteristics of the variables reflected in the existing data, this section proposes targeted practical improvement suggestions based on the distinct attributes of variables in “areas requiring key improvement,” “strength areas,” and “maintenance areas.” These suggestions offer valuable practical guidance for food enterprises, the packaging industry, and relevant authorities, specifically as follows:

Firstly, at the enterprise level: Three-dimensional synergistic optimization to facilitate consumer behavior transformation. For food enterprises, this study constructs a “key improvement—strength consolidation—maintenance optimization” three-dimensional synergistic optimization framework, integrating the variable influence effects reflected in the empirical data. Key improvements should focus on enhancing perceived trust levels by establishing a complete traceability loop for information and ensuring the authenticity and real-time nature of information regarding nutritional content and origin. Regular publication of third-party verification reports, coupled with simplification of operational procedures for freshness alerts and traceability inquiries, will augment consumer confidence in the information and functionality of smart packaging. The roles of core advantageous variables such as personal needs, social environment, and functionality should be consolidated by introducing stratified smart packaging products to accommodate diverse consumer demands, continuously optimizing the reliability of core functions like freshness monitoring and consumption reminders, and leveraging the demonstrative effect of groups to reinforce consumers’ normative perceptions. Variables related to experience, information, and environmental friendliness, categorized as maintenance variables, should be moderately optimized. This includes enhancing the user-friendliness of packaging interaction design, adopting biodegradable materials with clear recycling guidelines where cost-effective, and streamlining redundant information to highlight key health advisories, thereby progressively enhancing the driving effect of each variable.

Second, at the industry level: Design and technology, as dual drivers, will propel orderly industry transformation. For the food packaging industry, integrating the empirical conclusions of this study, we propose “design upgrading and technology empowerment” as the core to foster high-quality development. This involves establishing core design principles of “information transparency, functional reliability, and user-friendly experience,” eschewing superficial design tendencies, and focusing on developing practical intelligent packaging for core consumer use scenarios. We advocate for the introduction of lightweight intelligent sensing and identification technologies to reduce production costs and operational barriers while ensuring core functionality. Furthermore, specialized intelligent packaging will be developed for different categories such as fresh produce and pre-prepared meals to enhance product-scenario adaptability. Concurrently, we will promote the establishment and improvement of industry standards to regulate critical aspects like information transmission, functional testing, and eco-friendly material usage, guiding the industry towards refinement, standardization, and sustainability, thereby solidifying the foundation for industrial development.

Third, at the government level: Coordination of regulation and empowerment will create a favorable consumption environment. For regulatory and publicity departments, based on the consumer pain points and market realities identified in this study, we propose a healthy consumption support system of “regulatory constraints and knowledge empowerment.” This includes establishing a routine verification mechanism for the authenticity of information and reliability of functions of intelligent packaging, rigorously combating false traceability and exaggerated claims, and improving consumer rights protection mechanisms to maintain market fairness and consumer trust. Through diverse channels such as short videos and community lectures, we will precisely disseminate knowledge on the usage and health benefits of intelligent packaging, with a focus on practical skills like traceability inquiries and freshness interpretation, thereby enhancing consumers’ self-efficacy in healthy eating. Additionally, we will promote the development of specialized recycling systems for intelligent packaging by establishing collection facilities in communities and supermarkets, reducing obstacles to eco-friendly consumption and collaboratively facilitating the implementation of healthy and green consumption concepts, thereby creating a conducive external environment for the orderly penetration of the smart food packaging market.

## 6. Conclusions and Suggestions

This study, focusing on smart food packaging, constructed a chained mediating model based on the Theory of Planned Behavior and Perceived Value Theory: “Perceived Factors of Smart Food Packaging → Core TPB Constructs → Perceived Value Dimensions → Purchase Intention → Healthy Eating Behavior.” Hypotheses were validated using PLS-SEM and IPMA analyses, yielding core conclusions: the four perceived factors of smart food packaging (information, environmental friendliness, functionality, and experience) all exert a significant positive influence on the three core TPB constructs. Among these, the experience factor demonstrates the strongest driver of individual needs, challenging the traditional notion of “functionality over experience” and confirming the core value of convenient operational experience in enhancing consumers’ sense of behavioral control. The mediating paths from core TPB constructs to perceived value dimensions are significant, with the driver of individual needs being the strongest, indicating that consumers’ intrinsic needs for smart packaging are the core logical starting point for their value judgments and perceived trust. The differential impact strengths are explicitly clarified. Perceived value dimensions significantly and positively influence both purchase intention and healthy eating behavior, with perceived trust exerting the strongest influence on healthy eating behavior, thus confirming trust as the core nexus in the “value judgment-behavioral outcome” pathway. Purchase intention significantly and positively influences healthy eating behavior, validating the core tenet of the TPB that behavioral intention is a direct antecedent of actual behavior. IPMA analysis reveals that perceived trust is a key area for improvement, while individual needs, social environment, and functionality are strengths, and experience and environmental friendliness are maintenance areas. Environmental friendliness factors show the lowest overall scores. Furthermore, this study validated the effectiveness of the chained mediating model, clarified the internal mediating mechanisms, and provides support for research and practice in this domain.

Furthermore, this study still has certain limitations: the sample coverage is narrow, with insufficient representation from elderly populations and residents of lower-tier cities; moderating factors such as price and brand were not included; and cross-sectional data were used, precluding the capture of dynamic variable changes. Consequently, the conclusions drawn in this study regarding the association between purchase intention and healthy eating behaviors merely reflect the relationships within the current survey context and cannot be fully equated to the causal impact of intelligent packaging on actual healthy eating behaviors; its long-term dynamic mechanisms warrant further validation. Furthermore, the lack of differentiation across food categories results in insufficient specificity. Future research can achieve breakthroughs in four aspects: first, expanding sample coverage to encompass different demographic groups and regions, thereby enhancing the generalizability of the conclusions; second, refining the variable system by incorporating moderating factors like price and brand to deepen the model’s explanatory power; third, employing longitudinal tracking and experimental research to capture the dynamic transmission processes of variables; and fourth, differentiating food categories and integrating emerging technologies to refine smart packaging design and application, while also deepening interdisciplinary research to enrich research perspectives and provide more precise theoretical and practical support for industry development.

## Figures and Tables

**Table 1:** Definition of Variable Operability and Reference Scales.

Construct	Items	Source
Information factor (IF)	IF1 The intelligent packaging enables me to clearly understand the food traceability, production and logistics information. IF2 The information provided by the intelligent packaging, such as nutritional components and consumption tips, is comprehensive and reliable. IF3 The intelligent packaging can provide real-time feedback on key information such as food freshness and shelf life.	[2,3,4,5]
Environmental factor (EF)	EF1 I think the materials used in the intelligent packaging are more environmentally friendly and degradable. EF2 The intelligent packaging conforms to the development concept of green consumption and low-carbon environmental protection. EF3 Choosing the intelligent packaging can meet my personal pursuit of environmentally friendly consumption.	[29,30]
Functional factor (FF)	FF1 The intelligent packaging has practical functions such as freshness monitoring and quality warning. FF2 The intelligent packaging can effectively ensure the safety and freshness of food. FF3 The traceability and query functions of the intelligent packaging are convenient and practical.	[28]
Experiential factor (ETF)	ETF1 The intelligent packaging is easy to operate and the scanning query is convenient and fast. ETF2 The interface of the intelligent packaging is clear and the information is easy to read and understand. ETF3 The overall experience of using the intelligent packaging is smooth and comfortable.	[28,29]
Attitude (AT)	AT1 I have a positive and approving attitude towards the intelligent packaging of food. AT2 I think using the intelligent packaging is valuable and meaningful. AT3 I like and am willing to accept this new packaging form of intelligent packaging.	[12,47]
Social environment (SE)	SE1 Family and friends around me tend to purchase food with intelligent packaging. SE2 Public opinion and publicity advocate the use of intelligent packaging food. SE3 The healthy and environmentally friendly consumption atmosphere prompts me to choose the intelligent packaging.	[12,24]
Individual needs (IN)	IN1 I have the ability and conditions to easily use the various functions of the intelligent packaging. IN2 Using the intelligent packaging will not bring me operational difficulties or troubles. IN3 I can independently decide whether to purchase and use the food with intelligent packaging.	[14]
Perceived value (PV)	PV1 The safety and convenience benefits brought by the intelligent packaging are greater than its cost. PV2 Overall, the intelligent packaging has a high value for me. PV3 The intelligent packaging has a high cost-performance ratio and is worth purchasing.	[13,39,40,41]
Perceived trust (PT)	PT1 I believe the information displayed by the intelligent packaging is true and reliable. PT2 I trust the quality monitoring and safety guarantee functions of the intelligent packaging. PT3 I think the intelligent packaging can effectively protect the rights and interests of consumers.	[15,53]
Self-efficacy for healthy behaviors (SEHB)	SEHB1 I can better achieve healthy eating through the intelligent packaging. SEHB2 I am confident in choosing safer and healthier food through the intelligent packaging. SEHB3 With the intelligent packaging, I can better form healthy eating habits.	[55,56]
Purchasing intention (PI)	PI1 I will be willing to purchase food with intelligent packaging in the future. PI2 I will prioritize choosing food with intelligent packaging. PI3 I will recommend purchasing the food with intelligent packaging to others.	[53,57]
Healthy eating behavior (HEB)	HEB1 I will choose healthier food based on the intelligent packaging prompt. HEB2 I will avoid consuming food with an unsatisfactory prompt from the intelligent packaging. HEB3 The intelligent packaging helps me optimize my diet structure and form healthy eating habits.	[56,58]

**Table 4:** Common variance explanation (*n* = 566).

Element	Initial Eigenvalue			Extract the Sum of Squares and Load		
	Aggregate	Variable %	Cumulative %	Aggregate	Variable %	Cumulative %
1	14.268	39.633	39.633	14.268	39.633	39.633
2	2.891	8.030	47.663	2.891	8.030	47.663
3	1.067	2.964	50.627	1.067	2.964	50.627
4	0.933	2.592	53.219			
5	0.899	2.497	55.716			
6	0.872	2.423	58.139			
7	0.832	2.310	60.449			
8	0.800	2.221	62.671			
9	0.769	2.137	64.808			
10	0.748	2.077	66.885			
11	0.704	1.957	68.842			
12	0.696	1.934	70.775			
13	0.677	1.880	72.656			
14	0.654	1.816	74.471			
15	0.641	1.780	76.251			
16	0.620	1.722	77.974			
17	0.599	1.664	79.638			
18	0.572	1.589	81.227			
19	0.534	1.485	82.712			
20	0.521	1.448	84.160			
21	0.487	1.354	85.513			
22	0.459	1.275	86.788			
23	0.455	1.263	88.051			
24	0.433	1.204	89.255			
25	0.427	1.186	90.441			
26	0.412	1.144	91.585			
27	0.385	1.070	92.655			
28	0.380	1.055	93.710			
29	0.357	0.991	94.702			
30	0.351	0.974	95.676			
31	0.335	0.931	96.607			
32	0.306	0.849	97.456			
33	0.295	0.820	98.276			
34	0.271	0.754	99.030			
35	0.207	0.574	99.604			
36	0.143	0.396	100			

**Table 5:** Results of common latent factor approach (*n* = 566).

Indicator	Substantive Factor Loading (R1)	R1^2^	Method Factor Loading (R2)	R2^2^
IF1	0.880	0.7744	0.010	0.0001
IF2	0.881	0.776161	−0.021	0.000441
IF3	0.890	0.7921	0.011	0.000121
EF1	0.883	0.779689	0.028	0.000784
EF2	0.878	0.770884	−0.002	0.000004
EF3	0.881	0.776161	−0.027	0.000729
FF1	0.883	0.779689	−0.058	0.003364
FF2	0.871	0.758641	0.055	0.003025
FF3	0.891	0.793881	0.004	0.000016
ETF1	0.890	0.7921	−0.033	0.001089
ETF2	0.886	0.784996	−0.040	0.0016
ETF3	0.895	0.801025	0.072	0.005184
AT1	0.893	0.797449	0.100	0.010
AT2	0.900	0.810	−0.02	0.0004
AT3	0.884	0.781456	−0.082	0.006724
SE1	0.905	0.819025	0.018	0.000324
SE2	0.885	0.783225	−0.085	0.007225
SE3	0.891	0.793881	0.065	0.004225
IN1	0.876	0.767376	−0.079	0.006241
IN2	0.890	0.7921	0.047	0.002209
IN3	0.878	0.770884	0.031	0.000961
PV1	0.890	0.7921	−0.026	0.000676
PV2	0.896	0.802816	0.056	0.003136
PV3	0.889	0.790321	−0.031	0.000961
PT1	0.887	0.786769	−0.016	0.000256
PT2	0.897	0.804609	0.049	0.002401
PI3	0.896	0.802816	−0.033	0.001089
HEB1	0.894	0.799236	0.065	0.004225
HEB2	0.875	0.765625	−0.040	0.0016
HEB3	0.900	0.810	−0.026	0.000676
Average	0.892	0.795664	−0.059	0.003481

**Table 6:** Exploratory factor analysis result (*n* = 566).

Dimension	Items	KMO	Bartlett Sphere Test	Factor Loading	Commonality	Eigenvalue	Total Variation Explained %
IF	IF1	0.742	0	0.887	0.787	2.395	79.834
	IF2			0.897	0.804		
	IF3			0.896	0.804		
EF	EF1	0.736	0	0.894	0.799	2.374	79.146
	EF2			0.875	0.766		
	EF3			0.900	0.809		
FF	FF1	0.740	0	0.887	0.786	2.384	79.465
	FF2			0.891	0.793		
	FF3			0.897	0.805		
ETF	ETF1	0.740	0	0.894	0.800	2.379	79.286
	ETF2			0.889	0.790		
	ETF3			0.888	0.789		
AT	AT1	0.736	0	0.880	0.774	2.341	78.036
	AT2			0.881	0.776		
	AT3			0.889	0.791		
SE	SE1	0.734	0	0.882	0.778	2.326	77.539
	SE2			0.878	0.772		
	SE3			0.881	0.776		
IN	IN1	0.733	0	0.883	0.780	2.332	77.718
	IN2			0.871	0.758		
	IN3			0.891	0.793		
PV	PV1	0.740	0	0.891	0.793	2.379	79.298
	PV2			0.886	0.786		
	PV3			0.895	0.800		
PT	PT1	0.740	0	0.892	0.795	2.387	79.574
	PT2			0.900	0.810		
	PT3			0.885	0.782		
SEHB	SEHB1	0.740	0	0.905	0.818	2.397	79.884
	SEHB2			0.886	0.785		
	SEHB3			0.891	0.793		
PI	PI1	0.734	0	0.877	0.769	2.331	77.684
	PI2			0.889	0.791		
	PI3			0.878	0.771		
HEB	HEB1	0.741	0	0.890	0.792	2.385	79.490
	HEB2			0.896	0.803		
	HEB3			0.889	0.790		

**Table 7:** Convergent factor analysis results (*n* = 566).

Dimension	Items	Unstandardized	Standardize	SE	*p*-Value	AVE	CR
		Factor Loading	Factor Loading				
IF	IF1	1	0.827	-	-	0.698	0.874
	IF2	1.021	0.844	0.040	0		
	IF3	1.038	0.835	0.042	0		
EF	EF1	1	0.843	-	-	0.688	0.869
	EF2	0.953	0.809	0.039	0		
	EF3	0.999	0.836	0.039	0		
FF	FF1	1	0.830	-	-	0.692	0.871
	FF2	1.004	0.824	0.041	0		
	FF3	1.045	0.842	0.041	0		
ETF	ETF1	1	0.832	-	-	0.689	0.869
	ETF2	0.974	0.824	0.039	0		
	ETF3	0.986	0.834	0.039	0		
AT	AT1	1	0.817	-	-	0.671	0.860
	AT2	0.974	0.815	0.042	0		
	AT3	1.020	0.826	0.041	0		
SE	SE1	1	0.819	-	-	0.663	0.855
	SE2	0.932	0.813	0.039	0		
	SE3	0.930	0.811	0.039	0		
IN	IN1	1	0.811	-	-	0.666	0.857
	IN2	0.993	0.812	0.042	0		
	IN3	1.032	0.825	0.043	0		
PV	PV1	1	0.824	-	-	0.689	0.869
	PV2	0.970	0.822	0.040	0		
	PV3	0.990	0.844	0.039	0		
PT	PT1	1	0.847	-	-	0.694	0.872
	PT2	0.997	0.837	0.038	0		
	PT3	0.923	0.815	0.037	0		
SEHB	SEHB1	1	0.849	-	-	0.699	0.875
	SEHB2	0.948	0.816	0.038	0		
	SEHB3	0.976	0.843	0.037	0		
PI	PI1	1	0.802	-	-	0.665	0.856
	PI2	1.065	0.829	0.045	0		
	PI3	1.000	0.816	0.043	0		
HEB	HEB1	1	0.825	-	-	0.692	0.871
	HEB2	1.022	0.846	0.041	0		
	HEB3	1.005	0.825	0.041	0		

Note: 0.5 is the lowest standard for AVE and CR value > 0.7.

**Table 8:** Discrminant validty-Heterotrait ratio (*n* = 566).

Latent Variable	AT	HEB	IN	PI	PT	PV	SEHB	SE	ETF	IF	FF	EF
AT												
HEB	0.683											
IN	0.670	0.707										
PI	0.664	0.669	0.658									
PT	0.817	0.838	0.837	0.811								
PV	0.694	0.681	0.712	0.669	0.845							
SEHB	0.676	0.706	0.718	0.670	0.830	0.723						
SE	0.661	0.685	0.678	0.671	0.821	0.675	0.691					
ETF	0.686	0.716	0.695	0.667	0.832	0.699	0.673	0.690				
IF	0.691	0.689	0.669	0.691	0.830	0.691	0.696	0.687	0.689			
FF	0.687	0.707	0.676	0.656	0.836	0.711	0.693	0.680	0.699	0.679		
EF	0.689	0.694	0.690	0.659	0.824	0.697	0.681	0.687	0.687	0.710	0.702	

**Table 9:** HTMT inference (*n* = 566).

	Original Data (o)	Data Average (M)	5.0%	95.0%	Data Average (M)	Bias	5.0%	95.0%
HEB <-> AT	0.683	0.682	0.636	0.724	0.682	−0.001	0.637	0.725
IN <-> AT	0.670	0.670	0.624	0.714	0.670	0	0.623	0.714
IN <-> HEB	0.707	0.707	0.666	0.746	0.707	0	0.665	0.744
PI <-> AT	0.664	0.664	0.620	0.706	0.664	0	0.620	0.706
PI <-> HEB	0.669	0.668	0.622	0.711	0.668	−0.001	0.622	0.710
PI <-> IN	0.658	0.658	0.617	0.698	0.658	0	0.617	0.698
PT <-> AT	0.817	0.817	0.776	0.856	0.817	0	0.776	0.856
PT <-> HEB	0.838	0.838	0.799	0.874	0.838	0	0.798	0.872
PT <-> IN	0.837	0.837	0.796	0.876	0.837	0	0.796	0.876
PT <-> PI	0.811	0.812	0.769	0.85	0.812	0.001	0.766	0.847
PV <-> AT	0.694	0.693	0.646	0.738	0.693	−0.001	0.645	0.737
PV <-> HEB	0.681	0.681	0.637	0.721	0.681	0	0.638	0.721
PV <-> IN	0.712	0.712	0.669	0.752	0.712	0	0.669	0.752
PV <-> PI	0.669	0.668	0.624	0.709	0.668	0	0.622	0.710
PV <-> PT	0.845	0.845	0.797	0.889	0.845	0	0.797	0.889
SEHB <-> AT	0.676	0.676	0.631	0.717	0.676	0	0.629	0.716
SEHB <-> HEB	0.706	0.705	0.660	0.742	0.705	0	0.660	0.741
SEHB <-> IN	0.718	0.718	0.677	0.758	0.718	0	0.677	0.758
SEHB <-> PI	0.670	0.670	0.630	0.708	0.670	0	0.630	0.707
SEHB <-> PT	0.830	0.830	0.790	0.868	0.830	0	0.790	0.868
SEHB <-> PV	0.723	0.723	0.680	0.763	0.723	0	0.680	0.763
SE <-> AT	0.661	0.660	0.617	0.701	0.660	0	0.615	0.702
SE <-> HEB	0.685	0.685	0.640	0.725	0.685	0	0.640	0.725
SE <-> IN	0.678	0.678	0.635	0.719	0.678	0	0.633	0.718
SE <-> PI	0.671	0.671	0.629	0.711	0.671	0	0.629	0.711
SE <-> PT	0.675	0.675	0.631	0.715	0.675	0	0.631	0.712
SE <-> PV	0.675	0.675	0.633	0.717	0.675	0	0.631	0.714
SE <-> SEHB	0.691	0.691	0.649	0.729	0.691	0	0.648	0.728
ETF <-> AT	0.686	0.686	0.644	0.726	0.686	0	0.643	0.725
ETF <-> HEB	0.716	0.716	0.673	0.755	0.716	0	0.672	0.754
ETF <-> IN	0.695	0.695	0.655	0.733	0.695	0	0.654	0.732
ETF <-> PI	0.667	0.667	0.626	0.706	0.667	0	0.625	0.705
ETF <-> PT	0.832	0.831	0.785	0.875	0.831	−0.001	0.785	0.872
ETF <-> PV	0.699	0.699	0.656	0.738	0.699	0	0.655	0.737
ETF <-> SEHB	0.673	0.672	0.608	0.693	0.672	−0.001	0.606	0.691
ETF <-> SE	0.690	0.689	0.646	0.729	0.689	−0.001	0.646	0.728
IF <-> AT	0.691	0.691	0.646	0.732	0.691	0	0.646	0.732
IF <-> HEB	0.689	0.689	0.646	0.728	0.688	0	0.644	0.727
IF <-> IN	0.669	0.669	0.626	0.708	0.669	0	0.626	0.708
IF <-> PI	0.691	0.691	0.646	0.732	0.691	0	0.646	0.732
IF <-> PT	0.830	0.830	0.786	0.873	0.830	0	0.786	0.873
IF <-> PV	0.691	0.691	0.648	0.730	0.691	0	0.648	0.728
IF <-> SEHB	0.696	0.695	0.649	0.737	0.695	−0.001	0.646	0.734
IF <-> SE	0.687	0.687	0.644	0.726	0.687	0	0.642	0.728
IF <-> ETF	0.689	0.689	0.646	0.728	0.689	0	0.646	0.728
FF <-> AT	0.687	0.687	0.645	0.727	0.687	0	0.645	0.727
FF <-> HEB	0.707	0.707	0.666	0.746	0.707	0	0.665	0.747
FF <-> IN	0.676	0.676	0.629	0.719	0.676	0	0.629	0.719
FF <-> PI	0.656	0.656	0.609	0.699	0.655	0	0.609	0.696
FF <-> PT	0.836	0.836	0.797	0.870	0.836	0	0.797	0.87
FF <-> PV	0.711	0.712	0.669	0.750	0.712	0.001	0.666	0.747
FF <-> SEHB	0.693	0.692	0.644	0.734	0.692	−0.001	0.642	0.732
FF <-> SE	0.680	0.680	0.636	0.721	0.680	0	0.635	0.720
FF <-> ETF	0.699	0.699	0.657	0.739	0.699	0	0.655	0.741
FF <-> IF	0.679	0.679	0.636	0.717	0.679	0	0.635	0.718
EF <-> AT	0.689	0.689	0.647	0.729	0.689	0	0.647	0.729
EF <-> HEB	0.694	0.694	0.648	0.737	0.694	0	0.646	0.739
EF <-> IN	0.690	0.69	0.646	0.729	0.690	0	0.649	0.728
EF <-> PI	0.659	0.659	0.612	0.702	0.659	0	0.615	0.698
EF <-> PT	0.824	0.824	0.784	0.864	0.824	0	0.783	0.865
EF <-> PV	0.697	0.696	0.654	0.735	0.696	0	0.654	0.734
EF <-> SEHB	0.681	0.680	0.635	0.722	0.680	0	0.635	0.721
EF <-> SE	0.687	0.687	0.646	0.726	0.687	0	0.645	0.727
EF <-> ETF	0.687	0.687	0.644	0.728	0.687	0	0.647	0.725
EF <-> IF	0.710	0.710	0.666	0.753	0.710	0	0.668	0.752
EF <-> FF	0.702	0.701	0.655	0.742	0.701	−0.001	0.654	0.741

**Table 10:** Discriminant validity (Fornell–Larcker) (*n* = 566).

Latent Variable	AT	HEB	IN	PI	PT	PV	SEHB	SE	ETF	IF	FF	EF
AT	0.863											
HEB	0.822	0.852										
IN	0.792	0.812	0.852									
PI	0.809	0.809	0.795	0.871								
PT	0.812	0.828	0.819	0.818	0.872							
PV	0.806	0.802	0.815	0.801	0.804	0.860						
SEHB	0.813	0.823	0.827	0.817	0.833	0.803	0.874					
SE	0.807	0.817	0.801	0.809	0.825	0.807	0.820	0.871				
ETF	0.822	0.819	0.812	0.803	0.838	0.828	0.824	0.865	0.850			
IF	0.820	0.808	0.807	0.819	0.829	0.792	0.844	0.868	0.816	0.862		
FF	0.823	0.806	0.814	0.822	0.834	0.816	0.827	0.866	0.828	0.815	0.858	
EF	0.817	0.806	0.813	0.798	0.813	0.812	0.804	0.864	0.809	0.812	0.837	0.890

**Table 11:** Explanation of variance.

Dimension	R Square	R Square Adjusted	Q^2^ Predict	GOF
IN	0.839	0.838	0.600	0.753
SE	0.830	0.829	0.605	
HEB	0.828	0.827	0.606	
AT	0.843	0.842	0.609	
PV	0.830	0.829	0.623	
PT	0.838	0.837	0.720	
PI	0.832	0.831	0.551	
SEHB	0.832	0.831	0.599	

**Table 12:** Model fit measures.

Common Indices	d-ULS	d-G	SRMR	NFI
Criteria	<0.95	<0.95	<0.08	>0.8
Values	0.518	0.832	0.028	0.882

**Table 13:** Hypothesis model path relationship test.

Hypothesis	Path	VIF	β Co-Efficient	T Statistics	*p* Values	Decision
H1a	IF → AT	3.645	0.221	5.840	<0.001	Accept
H1b	IF → SE	3.645	0.272	6.481	<0.001	Accept
H1c	IF → IN	3.645	0.241	6.042	<0.001	Accept
H2a	EF → AT	3.976	0.235	5.670	<0.001	Accept
H2b	EF → SE	3.976	0.224	5.528	<0.001	Accept
H2c	EF → IN	3.976	0.247	6.238	<0.001	Accept
H3a	FF → AT	4.291	0.282	6.422	<0.001	Accept
H3b	FF → SE	4.291	0.228	5.012	<0.001	Accept
H3c	FF → IN	4.291	0.204	4.612	<0.001	Accept
H4a	ETF → AT	4.019	0.227	5.928	<0.001	Accept
H4b	ETF → SE	4.019	0.235	5.591	<0.001	Accept
H4c	ETF → IN	4.019	0.271	6.155	<0.001	Accept
H5a	AT → PV	3.115	0.323	8.548	<0.001	Accept
H5b	AT → PT	3.115	0.284	7.414	<0.001	Accept
H5c	AT → SEHB	3.115	0.247	6.365	<0.001	Accept
H6a	SE → PV	3.268	0.223	5.565	<0.001	Accept
H6b	SE → PT	3.268	0.360	9.059	<0.001	Accept
H6c	SE → SEHB	3.268	0.292	7.596	<0.001	Accept
H7a	IN → PV	3.102	0.407	10.604	<0.001	Accept
H7b	IN → PT	3.102	0.314	7.574	<0.001	Accept
H7c	IN → SEHB	3.102	0.414	10.678	<0.001	Accept
H8a	PV → PI	3.010	0.303	7.669	<0.001	Accept
H8b	PV → HEB	3.554	0.201	5.090	<0.001	Accept
H9a	PT → PI	3.470	0.344	7.756	<0.001	Accept
H9b	PT → HEB	4.174	0.316	6.718	<0.001	Accept
H10a	SEHB → PI	3.441	0.306	7.013	<0.001	Accept
H10b	SEHB → HEB	3.998	0.203	4.396	<0.001	Accept
H11	PI → HEB	3.936	0.236	5.079	<0.001	Accept

Note: A value of *p* < 0.05 indicates a significant feature.

## Data Availability

Data are contained within the article.
